# Culinary Nutrition Interventions for Those Living with and Beyond Cancer and Their Support Networks: A Systematic Review

**DOI:** 10.3390/curroncol33020076

**Published:** 2026-01-27

**Authors:** Marina Iglesias-Cans, Mizna Shahid, Lina Alhusseini, Killian Walsh, Laura Keaver

**Affiliations:** 1 Centre for Positive Health Sciences, RCSI University of Medicine and Health Sciences, D02 X0N1 Dublin, Ireland; marinaicans@rcsi.ie; 2 School of Medicine, RCSI University of Medicine and Health Sciences, D02 YN77 Dublin, Ireland; 3 RCSI Library, RCSI University of Medicine and Health Sciences, D02 YN77 Dublin, Ireland; 4 Department of Health and Nutritional Sciences, Atlantic Technological University, F91 YW50 Sligo, Ireland

**Keywords:** culinary nutrition, oncology nutrition, behaviour change, lifestyle intervention, life after cancer

## Abstract

People living with and beyond cancer often face ongoing challenges related to nutrition, wellbeing, and long-term health. Many individuals express a need for evidence-based, tailored dietary support, yet practical approaches to sustaining healthy eating behaviours remain limited. Culinary nutrition interventions, which integrate nutrition education with hands-on culinary skills, may help address these needs; however, their effects have not been systematically synthesised. This systematic review evaluates the impact of culinary nutrition interventions, delivered alone or in combination with physical activity or mental health components, on dietary intake, psychosocial and health-related outcomes, anthropometric measures, clinical and metabolic markers, and feasibility among individuals living with or beyond cancer. Following PRISMA guidelines, 18 studies were identified across PubMed, Scopus, EMBASE, CINAHL, and Web of Science (last searched in April 2025) and narratively synthesised. A total of 1173 participants were included, with sample sizes ranging from 4 to 190 participants per intervention. Interventions were well received and rated as highly acceptable, with strong engagement and minimal adverse effects. Across studies, statistically significant improvements were reported in dietary intake (7/13 studies), quality of life (4/5), mental health (5/6), self-efficacy (2/3), symptom management (3/4), self-reported cognitive health (1/1), food-related behaviours (2/2), selected anthropometric measures (4/8), and selected metabolic biomarkers (4/6). The evidence suggests that culinary nutrition interventions hold promise as supportive, behaviour-focused strategies aligned with oncology nutrition guidelines and responsive to patient needs. However, due to heterogeneity across interventions and outcomes, and variability in methodological quality as assessed using the Cochrane risk of bias tool, quantification of effects was not possible, limiting interpretation of the evidence. Further high-quality studies using comparable outcome measures and longer-term follow-up are needed to quantify the magnitude of effects, assess their durability over time, and inform the integration of culinary nutrition programmes into cancer care. This systematic review is registered under the PROSPERO ID CRD42024567041 and was funded by the RCSI Research Summer School Fund.

## Simple Summary

Life after cancer brings ongoing challenges related to nutrition, wellbeing, and long-term health. Many people living with and beyond cancer find it difficult to follow recommended healthy eating habits and need practical, supportive dietary guidance. Culinary nutrition interventions may offer a useful solution, as they combine nutrition knowledge with hands-on cooking skills, sensory experiences, behaviour change strategies, and social support. Previous studies suggest that cooking skill interventions can improve food literacy, build confidence around food choices, reduce food-related anxiety, and strengthen social connections and family involvement. This review examined the current scientific literature to assess the effects of culinary nutrition interventions on health-related outcomes for people living with and beyond cancer, as well as for their support networks. Overall, the findings show that these interventions, whether delivered alone or combined with physical activity or mental health components, are generally beneficial. Improvements were seen across most outcome areas, particularly those valued by the cancer community, such as dietary intake and quality of life. Although the exact size of these effects cannot be determined, this review highlights their potential and identifies priorities for future research.

## 1. Introduction

Global cancer rates are increasing, with an estimated 10 million cancer deaths in 2022 [1,2], though advances in screening and treatment have led to better survival rates [3]. According to the World Health Organisation (WHO) and the International Agency for Research on Cancer (IARC), there were approximately 53.5 million people worldwide who were alive within five years of a cancer diagnosis in the same year, 2022 [4]. Life after cancer comes with its own mental and physical health challenges; cancer treatments and their associated long-term effects can cause major changes in people’s lives, significantly increasing the likelihood of developing comorbidities such as cardiovascular disease, diabetes, and osteoporosis, affecting the quality of life and wellbeing of those affected by cancer and their close relatives [5].

Nutrition plays a central role in life after cancer. Evidence supports an increased risk of all-cause mortality with unhealthy dietary patterns and a reduced risk with healthier patterns among individuals affected by breast and colorectal cancer [6,7]. For breast cancer specifically, a meta-analysis of cohort studies provides strong evidence of an inverse association between high adherence to the Mediterranean diet and all-cause mortality [8]. Yet, people with a history of cancer often report the need for reliable food and nutrition information and resources to support their ongoing recovery and wellbeing [9,10]. Although nutrition and physical activity guidelines exist for individuals living with and beyond a cancer diagnosis, these recommendations are broad, not cancer-specific, and are largely adapted from general cancer prevention guidelines [6]. Moreover, a recent cross-sectional study by the American Cancer Society (n = 10,020) reported that 88% of respondents living with and beyond cancer did not adhere to the recommended healthy eating habits [11]. Establishing and sustaining healthy eating behaviours is often challenging after treatment; factors such as fatigue, stress, depression, limited knowledge and resources, unsupportive food environments, and changes in taste and appetite have been identified as the main barriers to maintaining healthy dietary behaviours [10,12,13]. Furthermore, many people affected by cancer report seeking nutrition information from external sources due to the lack of accessible dietary support within healthcare system settings, despite uncertainty about the reliability of these sources [9,14,15,16,17]. When advice is received, it is not always perceived as relevant to needs and preferences, and individuals frequently express a desire to receive evidence-based, tailored dietary support [9,14,15,17]. Together, these findings underscore a critical gap in post-treatment care and highlight the need for structured, accessible, and personalised nutrition resources and services for people living with and beyond cancer.

Nutrition education programmes for individuals living with and beyond cancer have demonstrated beneficial impacts. A recent systematic review of nutrition-related care in primary care settings found that nutrition interventions delivered by dietitians to people living with and beyond cancer produced meaningful changes in outcomes such as intentional weight loss and improved body composition [18]. Participants have reported improved nutrition knowledge, reduced obstacles to adopting healthier eating patterns, and greater confidence in implementing dietary advice [7,18,19,20]. Within nutrition programmes, culinary nutrition interventions combine nutrition education with hands-on cooking instruction to improve dietary behaviours and health outcomes [21]. Culinary nutrition interventions may be more effective than education alone because they merge nutrition knowledge with practical cooking skills, sensory experiences, behavioural strategies, and social support [21,22,23]. Both adult and child participants show improved dietary quality, greater fruit and vegetable intake, and healthier eating habits [22,23,24,25]. Cooking skill interventions have positive effects on food literacy, particularly improving confidence in healthy cooking with vulnerable, low-socioeconomic groups gaining more benefits [25,26]. Beyond dietary change and healthy cooking, culinary nutrition programmes build long-term skills fostering confidence, reducing food anxiety and strengthening social connections and family engagement [27].

Culinary interventions may be a promising approach to promote healthy eating behaviours and improve health outcomes for people living with and beyond cancer; however, evidence regarding their specific content, structure, and effectiveness remains limited. In particular, there is a lack of comprehensive synthesis on how culinary interventions influence health-related outcomes among people affected by cancer, a population with unique nutritional needs and behavioural challenges. While a scoping review has previously mapped existing studies, their content, and delivery methods, no systematic review yet has critically evaluated the quality and impact of these interventions [28]. This systematic review therefore aims to address this gap by providing an in-depth analysis of culinary intervention quality and their associated health outcomes in cancer survivorship.

The main aim of this study was to conduct a systematic review to answer the following research question: “What is the impact on health-related outcomes of culinary nutrition interventions for people living with and beyond cancer, as well as their support networks?”

## 2. Materials and Methods

This systematic review was carried out according to the Cochrane Handbook [29] and Preferred Reporting Items for Systematic reviews and Meta-Analyses (PRISMA) guidelines [30]. It is registered under the PROSPERO ID CRD42024567041.

### 2.1. Eligibility Criteria

#### 2.1.1. Inclusion and Exclusion Criteria

Quantitative and qualitative evaluations of randomised and non-randomised controlled culinary nutrition education interventions were reviewed,, including interventions with other components such as physical activity or mental health. As a prerequisite for inclusion, only studies targeting people of all ages affected by any type of cancer, as well as their support networks and carers, were included. The programmes could be group-based or individual, but must include a culinary component (at least one session) which could be delivered in real-time, through pre-recorded videos, or via written materials. Interventions could be delivered in person, over the phone, or online. The review included studies with or without a control or comparator. In terms of outcome measures, preferred outcomes identified by those living with and beyond cancer, such as diet quality and quality of life, were considered [31], along with other outcomes including dietary, psychosocial, clinical, metabolic, anthropometric, and feasibility. Culinary nutrition interventions that were not targeted at people living with and beyond cancer or their support networks were not included.

#### 2.1.2. Search Criteria

The online databases PubMed, Scopus, Excerpta Medica Database (EMBASE), Cumulative Index to Nursing and Allied Health Literature (CINAHL), and Web of Science were used to search the literature. The literature search was carried out by KW between January and April 2025. Only peer-reviewed articles written in the English language and published in the last 10 years were included. The search terms were refined several times to optimise article selection while maintaining search sensitivity, given the large volume of literature published on the topic of nutrition and cancer. The full search strategy for each database is presented in Supplementary Figure S1.

### 2.2. Study Selection

Titles and abstracts retrieved from the search were imported into Covidence systematic review software (Veritas Health Innovation, Melbourne, VIC, Australia). Two authors (MIC and LA) independently reviewed the search results and screened all publications. Initially, titles and abstracts were screened, and any conflicts were resolved through group discussion. Similarly, the full texts of selected articles were reviewed independently by the same authors, with disagreements also resolved through discussion. The selection process followed the established inclusion criteria. Protocol papers, reviews, editorial letters, and conference abstracts were excluded, as well as records for which neither the abstract nor full text could be accessed.

### 2.3. Data Extraction

A standardised data extraction format was followed for the extraction process, created in Microsoft Excel (Microsoft Office LTSC Professional Plus 2021, Version 2108) and presented here in table form. To gather the studies’ characteristics, the extracted data included author and programme name; place; participants, ethnicity, cancer type, timepoint (before, during, or after treatment), sample size, gender, and age; study design; main nutrition intervention components and specific culinary-related content; delivery, length, and frequency; intervention design, resources, theoretical frameworks, and co-design; and data collection timepoints and health-related outcome measures, including any quantitative non-significant and significant between- and within-group results reported. Two reviewers (MS and MIC) independently extracted data from the included studies. Any disagreement was settled via discussion with a third reviewer (LK).

### 2.4. Data Analysis

Since a meta-analysis was not possible due to the high diversity of interventions and outcome measures, studies’ results were narratively synthesised following the Cochrane Handbook and Synthesis Without Meta-analysis (SWiM) guidelines [29,32]. This method is intended to examine trends among studies and does not provide information about effect size or evaluate statistical significance; the summary results presented across the studies require careful interpretation.

### 2.5. Study Risk of Bias Assessment

The Risk of Bias 2.0 (RoB 2) tool for randomised studies [33] and the Risk of Bias in Non-randomised Studies of Interventions (ROBINS-1 V2) tool for non-randomised studies [34] were used to conduct a risk of bias assessment for the included studies in this review. The RoB 2 tool assessed randomised studies under five domains: bias arising from the randomisation process, bias due to deviations from intended interventions, bias due to missing outcome data, bias in the measurement of the outcome, and bias in the selection of the reported result. For each domain, and for the overall risk of bias of each study, the results were on a scale of low risk, some concern, or high risk of bias. The ROBINS-1 V2 tool assessed non-randomised studies under seven domains: risk of bias due to confounding, risk of bias in the classification of interventions, risk of bias in the selection of participants into the study, risk of bias due to deviations from intended interventions, risk of bias due to missing data, risk of bias arising from measurement of the outcome, and risk of bias in the selection of the reported result. For each domain, and for the overall risk of bias of each study, the results were on a scale of low, moderate, serious, or critical risk of bias. The risk of bias was independently assessed by two reviewers (MS and MIC). Any disagreement was settled via discussion with a third reviewer (LK).

## 3. Results

### 3.1. Characteristics of the Studies

**Figure 1:**
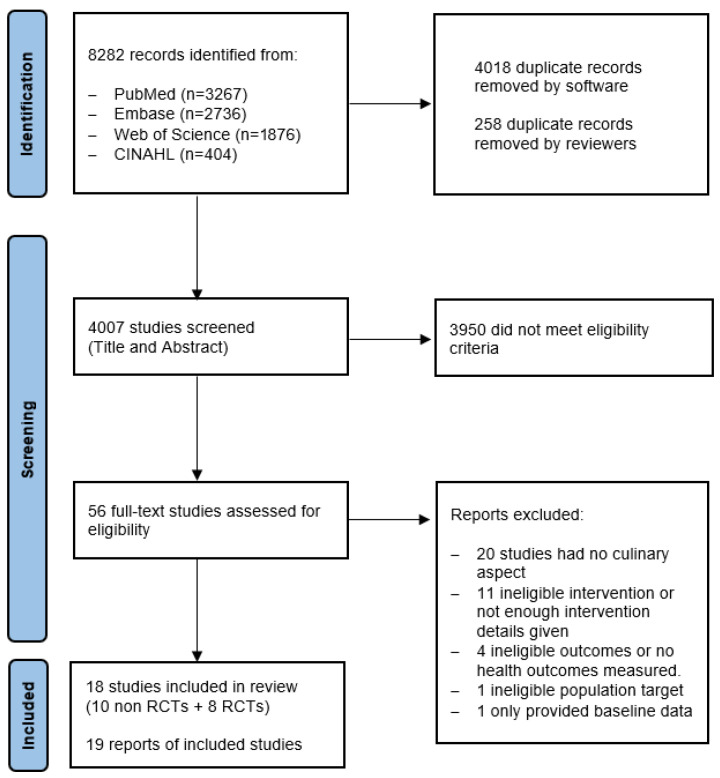
Flowchart detailing the study selection.

A total of 8283 records were retrieved from five databases, with 4265 of those titles included in the screening after duplicates were removed by the software. An additional 256 duplicates were manually removed by the reviewers, leaving 4008 for screening. Of these, 19 articles (reporting on 18 studies) were selected and included in the systematic review; a study flow diagram is presented in Figure 1. A total of 1173 participants were included, with sample sizes ranging from 4 to 190 participants per intervention.

The characteristics of the studies and their interventions are described in Table 1 for both non-randomised and randomised controlled studies. Nine articles (44%) reported on 8 randomised controlled trials (RCTs) (mean sample size = 31.8, SD = ±14.0; range 15–60) [35,36,37,38,39,40,41,42,43], and 10 studies (56%) were non-randomised controlled trials (non-RCSTs) (mean sample size 46.3, SD ±33.5; range 4–102) [44,45,46,47,48,49,50,51,52,53]. Only two non-RCT studies included a control group [48,52]. Of the 19 articles, 13 were quantitative (68%) [35,37,39,40,41,42,43,45,48,49,50,51,53] and 6 were mixed-methods (32%) studies [36,38,44,46,47,52]. The majority of the studies were conducted in the USA (n = 14, 78%) [35,36,37,38,40,41,42,43,44,45,49,50,51,53], followed by Canada (n = 2, 11%) [46,47], Israel (n = 1, 5%) [52], Japan (n = 1, 5%) [48], and Spain (n = 1, 5%) [39]. All studies were conducted in adult populations. Ten studies (56%) focused on female participants [35,36,37,38,39,41,42,43,46,50]. Eight studies included both men and women; however, in six of these studies, women constituted 75% or more of the sample [40,44,47,48,49,51,52]. The average sample age ranged between 50 and 64 years old. One study did not report the gender and age of its participants [45]. Nine studies (50%) involved participants with a history of breast cancer [35,36,37,38,39,41,42,43,50], seven (39%) included participants with any cancer type [40,44,45,47,49,51,52], one (6%) head and neck cancer [53], one (6%) digestive cancer [48], and one (6%) gynaecological cancers [46]. Three studies (17%) allowed caregiver participation in the programme [46,47,53]. Fifteen studies (83%) did not target any specific ethnicity [35,37,38,39,40,41,44,45,46,47,48,49,50,51,52], one study (6%) was conducted in Hispanic populations [42,43], one (6%) in African American populations [36], and one (6%) in Caucasian adults [53]. The majority of the studies (n = 15, 83%) were conducted post-treatment [35,36,38,40,41,42,43,44,45,46,47,48,49,50,51,53], two interventions (11%) took place during treatment [37,39], and one (6%) during and post-treatment [52].

### 3.2. Characteristics of Interventions

Half of the interventions (n = 9) included only a single culinary/nutrition-related component [35,40,42,43,45,46,47,48,49,53], and the other half were multicomponent. Eight interventions (44%) included a physical activity element [36,37,38,39,41,44,50,51], and four (22%) had a psychosocial aspect [36,50,51,52]. Kondo et al. (2023) was the only intervention that did not report including nutrition education alongside the culinary cooking component [48]. Fourteen studies (77%) were conducted in-person in group settings [35,36,37,38,39,40,42,43,44,45,46,47,51,52,53]. Of these, five interventions included a hybrid format and followed participants up with phone calls and/or emails [35,36,37,47,51], and one included online coaching [44]. Three interventions were delivered fully online [41,49,50], and one intervention was one-to-one, in-person [48]. Fifteen interventions (83%) included registered dietitians or nutritionists as part of the delivery team [36,38,39,40,41,42,43,46,47,48,49,50,51,52,90], while 14 studies (77%) included chefs and culinary professionals [35,38,40,41,42,43,44,46,47,50,51,53]. Raber’s programme “Cooking after cancer” was delivered by trained peer facilitators with a personal connection to cancer [45], while Sheean’s RCT “Everyday counts” was delivered by both health and athletic coaches [37]. Other programme facilitators included mental health professionals, physiotherapists, behavioural health specialists, physicians, social workers, and occupational therapists. Interventions ranged from one-off 30 min sessions to 24 week programmes; see Table 1 for details.

All interventions, except for one [48], reported basing their educational content on specific nutrition guidelines and recommendations: the American Institute for Cancer Research (AICR) guidelines [38,40,41,42,43,44,45,49,50,52], the American Cancer Society (ACS) guidelines [36,37,38,41,42,43], the Mediterranean diet guidelines [35,50,51], the American Heart Association and American College of Cardiology (AHA/ACC) Guidelines [44], the Spanish Society of Community Nutrition Guidelines [39], the World Health Organisation’s Nutrient Intake Goal [53], guidelines for fatigue reduction through diet for people affected by cancer [47], nutrition, cancer, and gastrointestinal (GI) complication guidelines [46], garden-based lifestyle interventions [44], and the World Health Organisation physical activity guidelines [39]. Fifteen studies based their interventions’ format and delivery on established behaviour change techniques, such as social learning theory and social cognitive theory [36,37,38,40,41,42,43,46,47], the transtheoretical model of health behaviour change [40,42,43,53], experiential learning theory [46,47,48], self-determination theory [41,44], Contento’s model [42,43], the theory of planned behaviour [36], stages of change [35], lifestyle behaviour change frameworks [51], and other behaviour change techniques [40,46,47,50,52]. Three programmes did not base their interventions on health behavioural theories [39,45,49]. Finally, seven interventions engaged multiple stakeholders through co-design and community-based participatory research methods: community-led co-design [37,42,43], stakeholder-led co-design [40], and participant-led co-design [48,50,52,53].

### 3.3. Nutrition Component of the Interventions

The nutrition education content of most interventions included a combination of nutrition education sessions [35,36,37,38,39,40,41,42,43,44,45,46,47,49,50,51,52,53] alongside either cooking demonstrations [35,36,39,40,42,43,44,49,50,51,53], hands-on cooking lessons [37,41,46,47,52], or both [38,45]. Kondo et al. (2023) delivered hands-on cooking lessons and did not report a nutrition education component, even though the intervention was facilitated by a dietitian and an occupational therapist [48].

The main topics addressed general healthy eating, food groups, and nutrients [35,36,37,38,39,40,41,42,43,44,45,46,47,49,50,51,52,53]; meal planning, recipes, portion size, and meal preparation [35,36,37,38,39,40,41,42,43,45,46,47,49,50,52,53,60,90]; food shopping, food labels, and eating out [35,38,39,40,41,42,43,45,46,48,50,51,52,53]; psychosocial aspects of food such as mindful eating, food anxiety, emotional wellbeing, meal sharing, and eating together [39,40,41,42,43,48,50,52,53]; barriers to healthy eating, goal setting, coaching, and behaviour change strategies [35,36,37,40,44,47,50,51,52,53]; cancer-related food taste changes [35,40,42,43,51,52,53], and other nutrition-related modifiable risk factors for cancer [36,38,44,45,46,47,48,50]. Several interventions focused on specific dietary approaches, including whole food plant-based diets [37,40,44,45], Mediterranean-style diets [35,50,51], and anti-inflammatory diets [35,49], as well as diets addressing cancer-related fatigue [47] and cancer-related GI issues [46,48].

With regard to the culinary nutrition component of the interventions, in most studies, the cooking sessions complemented the nutrition education content delivered. Cooking demonstrations and hands-on sessions were facilitated by chefs (n = 8, 44%) [35,38,42,43,44,46,47,51], culinary specialists (n = 2, 11%) [40,50], or both (n = 2, 11%) [41,53]. Six studies did not include a culinary or chef professional in their delivery team (33%) [36,37,39,45,48,49,52]. In four studies, participants were invited to select the recipes to prepare [48,50,52,53], while three studies culturally tailored the cooking sessions to align with participants’ dietary traditions [36,42,43,52]. The culinary sessions typically involved recipe preparation, ingredient selection, increasing the inclusion of healthier options such as fruits, vegetables, herbs, and spices, and the development of practical cooking skills (e.g., knife skills and equipment use) [35,36,37,38,40,41,42,43,44,46,47,48,49,50,51,52,53]. Five interventions focused on Mediterranean-style cooking (28%) [35,49,50,51,52], six on preparing and sharing meals together (33%) [35,38,40,41,42,43,45,51,52,53], and six on introducing healthier alternatives (for example, enhancing sweetness without added sugar, or flavour without added salt, or increasing the fibre content) (33%) [42,43,47,49,50,51]. Other sessions emphasised cooking methods that promote convenience and taste (50%) [35,38,40,42,43,45,47,51,52,53], have anti-inflammatory properties (17%) [35,47,49], address cancer-related dietary challenges (39%) [35,38,39,44,45,46,47], utilise garden produce (6%) [44], or incorporate friendly cooking competitions (6%) [38]. The culinary content was only briefly described with little detail in four interventions (22%) [36,38,39,48].

### 3.4. Outcome Measures

Table 2 describes a summary of the outcome measures and results for each study. Across the studies included, outcome measures spanned across seven key areas: dietary intake, anthropometric measures, psychosocial wellbeing, health-related outcomes, clinical and metabolic measures, feasibility, and qualitative outcomes. Dietary intake was assessed in 13 studies (72%) using self-reported tools, including dietary recall [35,36,37,39] and questionnaires [35,38,40,44,45,49,52,53]. Psychosocial and behavioural outcomes were assessed in 12 studies (67%) and included quality of life (n = 5, 28%) [37,40,44,51,52]; perceived stress, anxiety, depression, and subjective wellbeing (n = 6, 33%) [37,40,41,48,51,52]; self-efficacy and confidence in symptom management (n = 6, 28%) [36,40,44,46,47]; food-related behaviours (n = 2, 11%) [50,52]; lifestyle behaviours and risks factors (n = 2, 11%) [37,44]; emotional support (n = 1, 6%) [40]; and health literacy (n = 1, 6%) [38]. Eight studies additionally assessed health-related outcomes such as fatigue and energy levels (n = 3, 17%) [37,40,47], physical functioning (n = 3, 17%) [39,47,48], physical activity (n = 2, 11%) [36,44], and cognitive function (n = 1, 6%) [49]. Eight studies measured anthropometric outcomes; these were typically measured via weight and body mass index (BMI) (n = 8, 39%) [37,38,39,41,42,43,44,51] and waist circumference (n = 6, 33%) [37,39,41,42,43,44,51]. Clinical and metabolic outcome measures were included in six studies [36,37,39,42,44,51]; clinical measures included resting heart rate (n = 1, 6%) [39] and blood pressure (n = 3, 17%) [39,44,51], while metabolic outcomes comprising cardiometabolic fitness—VO_2_ max (n = 1, 6%) [36], lipid markers (n = 3, 17%) [39,44,51], glycaemic markers (n = 2, 11%) [44,51], inflammatory markers (n = 3, 17%) [42,44,51], nutrient/antioxidant markers (n = 2, 11%) [42,44], cellular energetics (n = 1, 6%) [37], and epigenetic markers (n = 1, 6%) [42]. Five studies evaluated the feasibility of the intervention alongside its effectiveness (28%) [38,39,44,46,47]. Qualitative outcomes were reported in six studies using focus groups, interviews, or questionnaires (33%) [36,37,38,44,46,47].

#### 3.4.1. Dietary Intake

Thirteen studies reported dietary intake outcomes; however, outcome measures were heterogeneous. Fruit and vegetable (F/V) intake was assessed in six studies. Greenlee et al. (2015, 2016) reported significant increases in F/V intake in the intervention arm versus control, maintained at 6- and 12-month follow-up (+2 and +2.3 F/V servings, respectively) [42,43]. Similarly, Greenlee et al. (2024) observed an increase in daily servings of F/V after the intervention (+1.5) [41]. In contrast, Miller et al. (2020), Morato-Martínez et al. (2021), Sheean et al. (2021), and Parekh et al. (2018) found no significant between-group differences in F/V intake [37,38,39,40]. Miller et al. (2020) and Parekh et al. (2018) also assessed nutrition knowledge, skills, and confidence. In the first study, nutrition knowledge improved significantly at both week 9 and 15 (+0.8 and +0.6), while nutrition confidence (+0.7) and skills (+0.4) showed significant improvement by week 15 [40]. Fat intake was assessed in three studies; Greenlee et al. (2015, 2016) observed a significant reduction in percent calories from total fat (−7.1%) and saturated fat (−3.8%) at 3 months, and non-significant changes at follow-up [42,43]. Similarly, Greenlee et al. (2024) recorded a reduction in fat intake post-intervention (−10 g of total fat and −4.6 of saturated fat) [41]; and Sheppard et al. (2016) observed non-significant changes in total fat, % energy from fat, and fibre intake [36].

Zuniga et al. (2019) assessed adherence to a Mediterranean diet using three-day food records and observed a significant increase in adherence to Mediterranean diet guidelines in the intervention arm (22.5% improvement). Improved adherence was noted for three specific guideline components: consuming ≥3 servings of fish or shellfish per week, reducing red meat intake to <1 serving per day, and limiting consumption of commercial sweets and baked goods to <3 times per week [35]; moreover, use of spices/herbs increased by 146.2%. Both Zuniga et al. (2019) and Greenlee et al. (2024) reported a significant reduction in total calorie intake in the intervention group (+195.5 kCal and −170 kCal, respectively) [35,41]. Jackson et al. (2024) further evaluated dietary intake using the Dietary Inflammatory Index (DII) and found a significant decrease (−2.7) in the inflammatory potential of the intervention group’s diet [49]. Barak-Nahum et al. (2016) also reported significant improvements, as measured by the Intuitive Eating Scale and Food Choice questionnaires (+13.5) [52]. Spees et al. (2019) observed significant improvements in dietary intake using the Healthy Eating Index (HEI) (+5.2) [44]. Parekh’s and Sheean’s studies achieved high dietary intake scores at baseline with limited potential for further improvement after the intervention [37,38]. Raber et al. (2022) [45] and Allen-Winters et al. (2020) [53] also evaluated dietary intake and found no significant changes. Overall, findings were mixed across single-component and multicomponent interventions, as well as between randomised and non-randomised controlled trials, with both study designs demonstrating significant and non-significant results.

#### 3.4.2. Quality of Life

Barak-Nahum et al. (2016) [52], Golubić et al. (2018) [51], Miller et al. (2020) [40], Sheean et al. (2021) [37], and Spees et al. (2019) [44] reported on quality of life, although different validated instruments were used. Sheean et al. (2021) reported statistically significant pre–post improvements in physical (+1.6), emotional (+1.4), and functional wellbeing (+1.2) measured using the FACT instrument [37]. Using the QOL-CSV, Spees et al. (2019) observed statistically significant improvements across physical, psychological, and spiritual quality-of-life domains (+16) [44]. Barak-Nahum et al. (2016) reported significantly higher health-related quality of life and affective wellbeing in the intervention group assessed using the SF-12 (+13.23), higher subjective positive affect (+40.6), and lower negative affect (−28) [52]. Golubić et al. (2018) identified statistically significant improvements in physical (+62%), mental (+51%), and overall health-related quality of life (+54%), measured using the VR-12 [51]. In contrast, Miller et al. (2020) found no statistically significant changes in quality of life when assessed using the FACT-G7 [40].

#### 3.4.3. Other Psychosocial Outcomes and Health-Related Outcomes

Six studies assessed mental health outcomes such as perceived stress, anxiety, depression, and subjective wellbeing. Miller et al. (2020) reported no significant improvements in psychological distress [40]. Golubić et al. (2018) found significant improvements in perceived stress (−20%), assessed using the Perceived Stress Scale (PSS) [51]. Greenlee et al. (2024) noted no significant changes in anxiety scores assessed via the Patient-Reported Outcomes Measurement Information System (PROMIS) questionnaire [41]. Sheean et al. (2021) reported mixed results with the Hospital Anxiety and Depression Scale (HADS): significant reductions in depression were observed in the intervention group compared with the control (−0.9), anxiety decreased significantly in the control group (−1.2), and perceived stress decreased significantly for participants in both groups, with a higher decrease for the intervention group (−2.8) [37]. Kondo et al. (2023) also assessed anxiety and depression using the HADS and reported significant improvements only in the intervention group, with intergroup comparisons indicating additional gains for both anxiety (−4) and depression (−6) [48]. Barak-Nahum et al. (2016) found significant improvements in subjective wellbeing, lower subjective wellbeing’s negative affect (+28), and positive affect (+41), measured using the Subjective Wellbeing Scale [52].

Regarding food-related behaviours, Huang et al. (2023) assessed mindfulness while eating via the Mindful Eating Questionnaire (MEQ) and found a significant increase in all MEQ subscale scores except for the distraction subscale (scale mean change 0.12) [50]. Similarly, Barak-Nahum et al. (2016) [52] reported a positive effect of the culinary intervention on intuitive eating and food choices, as measured by the Intuitive Eating Scale. Participants in the intervention group showed that intuitive eating, in general, increased over time: unconditional permission to eat (+25.1), eating for physical rather than emotional reasons (+22.9), and reliance on hunger and satiety cues (+37.3). Moreover, higher healthy food choice scores were associated with better health-related quality of life and positive affect, and with lower negative affect, independent of intervention group [52].

Pritlove et al. (2024) [46], Sheppard et al. (2016) [36], and Spees et al. (2019) [44] assessed the self-efficacy of participants, with Pritlove et al. (2024) [46] finding significant improvement in self-efficacy with regard to managing GI side effects. Qualitative findings also supported this result, with the improvements in knowledge and confidence providing an increase in empowerment and self-efficacy for some participants [46]. Sheppard et al. (2016) found that participants had high perceived control in achieving and maintaining their dietary and physical activity goals (overall satisfaction, 86%) [36], while Spees et al. (2019) noted non-significant changes for self-efficacy [44].

Symptom management was assessed by Miller et al. (2020) [40], Sheean et al. (2021) [37], Pritlove et al. (2020) [47], and Pritlove et al. (2024) [46]. Pritlove et al. (2020) found that fatigue scores significantly improved and were maintained across the full follow-up period (from baseline to week 9 and week 12, +5 and +7.75) [47]. Sheean et al. (2021) found that fatigue improved for the intervention group (+1.6) [37], and Miller et al. (2020) found no significant changes in fatigue symptoms [40]. Miller et al. (2020) observed that perceived control over the cancer trajectory remained relatively stable in both groups [40]. Pritlove et al. (2024) reported statistically significant improvements in bowel-related and GI symptoms post-intervention [46], while Sheean et al. (2021) observed that participants in the intervention group experienced meaningful improvements in both breast cancer-related symptoms (+6.8) and endocrine symptoms (+8.7) [37]. Jackson et al. (2024) [49] assessed cognitive function using the Functional Assessment of Cancer Therapy–Cognitive Function (FACT-Cog) tool and reported significant improvements in FACT-Cog subscores following the intervention: perceived cognitive impairment (Δmedian −38), comments from others (Δmedian −16), and quality of life (Δmedian −10).

#### 3.4.4. Anthropometric Measures

Eight studies reported anthropometric outcomes, assessed using BMI, body weight, or waist circumference [36,37,38,39,41,42,43,44,51]. Findings varied across studies. Morato-Martínez et al. (2021) observed significant decreases in body weight (−1.87 kg) and BMI (−0.61 kg/m^2^) in the intervention group [39], while Parekh et al. (2018) also reported a significant reduction in age-adjusted BMI (−0.69 kg/m^2^) [38]. Golubić et al. (2018) found significant reductions in BMI (−2.4 kg/m^2^), weight (−7%), and waist circumference (−6.6 cm) in the intervention arm [51]. Similarly, Spees et al. (2019) observed significant reductions in body weight (3.9 kg), BMI (−1.5 kg/m^2^), and waist circumference (−5.5 cm) [44]. In contrast, Greenlee et al. (2015, 2016), Greenlee et al. (2024), Sheppard et al. (2016), and Sheean et al. (2021) reported no significant changes in anthropometric measures [36,37,41,42,43,72]. The results differed across randomised and non-randomised controlled trials; significant improvements in anthropometric outcomes were observed in four multicomponent interventions that combined a physical activity component with a culinary or nutrition element [38,39,44,51]. Three other multicomponent interventions showed no significant changes [36,37,41], and one single-component intervention likewise reported no significant differences [42,43].

#### 3.4.5. Clinical and Metabolic Outcomes

Six studies reported on clinical and metabolic measures [36,37,39,42,44,51]. Golubić et al. (2018) [51], Spees et al. (2019) [44], and Morato-Martínez et al. (2021) [39] reported on blood pressure changes; Spees et al. (2019) observed a significant improvement in systolic blood pressure (−9.5 mmHg) [44], while Morato-Martínez et al. (2021) and Golubić et al. (2018) found no significant changes [39,51]; even though systolic and diastolic blood pressure did not reach statistical significance in Golubić’s Lifestyle180^®^ study, participants reported an overall decrease in their use of blood pressure medications, 30% of participants reduced BP medications, while 15% increased their medication [51]. Morato-Martínez et al. also found no changes in heart rate [39]. Regarding cardiometabolic fitness, Sheppard et al. (2016) examined changes in VO_2_ max and observed non-significant changes [36]. Morato-Martínez et al. (2021) [39], Golubić et al. (2018) [51], and Spees et al. (2019) [44] examined lipid profiles. Morato-Martínez et al. (2021) observed a significant reduction in LDL cholesterol in the intervention group at month 6 and 12 (−35.29 mg/dL and −12.5 mg/dL), as well as total cholesterol (−29.29 mg/dL and −32.92 mg/dL) with non-significant changes for triglycerides and HDL cholesterol [39]. Golubić et al. (2018) reported significant improvements in triglycerides (−23.0 mg/dL) and HDL-C (+3.3 mg/dL) and non-significant changes in LDL cholesterol; some participants decreased the use of lipid-lowering medication (12% reduced vs. 3% increased) [51], whereas Spees et al. (2019) found significant reductions in total cholesterol (−6%) and triglycerides (−14%) [44].

Greenlee et al. (2016), Morato-Martínez et al. (2021), Golubić et al. (2018), and Spees et al. (2019) assessed glycaemic markers [39,42,44,51]. Golubić et al. (2018) reported significant improvements in fasting insulin (−4.2 mU/mL) and insulin sensitivity (−1.5), and non-significant changes were observed in fasting plasma glucose [51]. In contrast, Spees et al. (2019), Greenlee et al. (2016), and Morato-Martínez et al. (2021) found no significant changes in insulin or fasting plasma glucose [39,42,44]. Golubić et al. (2018), Greenlee et al. (2016), and Spees et al. (2019) examined inflammatory markers [42,44,51]. Greenlee et al. (2016) examined inflammatory markers including interleukin-1 alpha (IL-1α), interleukin-10 (IL-10), tumour necrosis factor-alpha (TNF-α), and high-sensitivity C-reactive protein (hs-CRP), and found no significant changes [42]. In contrast, Spees et al. (2019) reported a significant decrease in hs-CRP levels (−28%) in the intervention group, as well as a reduction in Insulin-like Growth Factor Binding Protein 3 (IGFBP-3) (−5%) [44]. Golubić et al. (2018) also observed significant reductions in c-CRP (−1.3 mg/L) [51]. Greenlee et al. (2016) [42] assessed antioxidant status by measuring plasma carotenoids and found that participants in the intervention group achieved a significantly greater percent decrease in plasma lutein at both month 6 and 12 (+13.8% and +20.4%, respectively); no significant changes were observed in blood levels of lycopene, α-carotene, β-carotene, β-cryptoxanthin, and retinol [42]. Spees et al. (2019) reported significant increases in plasma carotenoid concentrations in the intervention group with total dietary carotenoids increasing by 66% and total plasma carotenoids by 35% [44]. Sheean et al. (2021) observed no changes in spare respiratory capacity [37], a measure of mitochondrial function and the cell’s ability to respond to increased energy demand [92]. Greenlee et al. (2016) observed no significant changes in DNA methylation [42]. Five out of the six studies reporting on clinical and metabolic measures were multicomponent [36,37,39,44,51], and no differences were present between RCTs [36,37,39,42] versus nRCTs [44,51] in terms of achieving significant results.

#### 3.4.6. Feasibility and Qualitative Outcomes

Spees et al. (2019) [44], Pritlove et al. (2020) [47], Pritlove et al. (2024) [46], and Parekh et al. (2018) [38] looked at the feasibility of interventions. Spees et al. (2019) [44] found that attendance and engagement were strong; participants attended 90% of education sessions and 59% of harvest weeks, with most completing at least 80% of intervention weeks. Use of the web portal (90%) and tele-motivational interviewing (59%) was high, and email was the preferred communication mode. Acceptability was excellent, with 93% rating the programme very positively. Participants valued the group education sessions most, reported meaningful health benefits and community support, and overwhelmingly indicated improvements in diet and physical activity, with nearly all planning to apply what they learned [44]. Pritlove et al. (2020) [47] assessed feasibility via qualitative interviews. Common themes regarding the feasibility of the intervention included that most participants found the programme length and frequency to be appropriate, that recipes and culinary strategies were easy to understand and implement, and that the programme was flexible regarding scheduling [47]. Pritlove et al. (2024) [46] also found similar themes; qualitative interviews reflected strong perceptions of feasibility, with modest recruitment (32%) but optimal retention (72%), suggesting a need to explore alternative formats. Participants reported high satisfaction and perceived utility of the program. Common recommendations included offering more in-class sessions and expanding the programme to enhance engagement and sustain participation over time [46]. Recruitment targets were achieved within six months in Parekh’s study (2018) [38] and session attendance was strong, with over half of participants attending all sessions. Qualitative feedback indicated high acceptability, with participants valuing the hands-on cooking activities and overall programme structure.

Six studies explored qualitative outcomes. Across these studies, key themes included participant satisfaction with the sessions, perceived benefits, and suggestions for programme enhancement. Parekh et al. (2018) reported that participants viewed the sessions as valuable and appreciated the supportive group environment [38]. In Spees et al. (2019), participants described reductions in medication use and reported experiencing fewer challenges with healthy eating following the intervention [44]. Both Sheppard et al. (2016) and Pritlove et al. (2020, 2024) noted high levels of satisfaction among participants [36,46,47]. Regarding recommendations for improvement, participants in Parekh et al. (2018) suggested incorporating booster sessions and providing information on tailored diets [38]. Those in Sheppard et al. (2016) proposed more opportunities for interaction with peers and facilitators, along with greater contextual tailoring of the programme [36]. Participants in Pritlove et al. (2020) recommended a multi-tiered structure based on participants’ skill levels and more one-to-one consultations [47], while those in Pritlove et al. (2024) expressed interest in additional in-person sessions and programme expansion [46]. Similarly, participants in Sheean et al. (2021) highlighted the value of expanding the intervention and offering more ongoing support [37]. No consistent differences in qualitative outcomes were observed between single- and multicomponent interventions or between randomised and non-randomised study designs.

### 3.5. Risk of Bias

For the nine RCTs included in this study, the overall risk of bias was high in three studies [37,38,40], and the remaining six studies had some concerns [35,36,39,41,42,43]. Risk of bias arising from the randomisation process was low in three studies [37,42,43], and of some concern in six studies [35,36,38,39,40,41]. Bias due to deviations from intended interventions was of some concern in all nine studies [35,36,37,38,39,40,41,42,43]. Bias due to missing outcome data was low risk in four studies [37,41,42,43], of some concern in three studies [35,39,40], and high in two studies [36,38]. Risk of bias in the measurement of outcome was low in one study [42], of some concern in six studies [35,36,38,39,41,43], and high in two studies [37,40]. Risk of bias in the selection of reported results was low in one study [41], and of some concern in the remaining eight studies [35,36,37,38,39,40,42,43]. For the 10 non-RCTs, the overall risk of bias was critical in eight studies [44,45,46,47,49,50,51,53] and moderate in two studies [48,52]. The risk of bias was critical in eight studies due to failure to control for confounding. Further details of the risk of bias assessments are provided in the Supplementary Materials.

## 4. Discussion

To our knowledge, this is the first systematic review to present a consolidated summary of the effects of culinary nutrition interventions on the health and wellbeing of people living with and beyond cancer. Eighteen programmes published in the literature within the past 10 years were deemed eligible. Despite considerable heterogenicity in intervention content and outcome measures, a consistent pattern emerged across interventions indicating generally positive effects on participants’ health and wellbeing. Our findings indicate that both culinary nutrition-only interventions and those combining nutrition with physical activity and/or mental health components have a positive impact on dietary intake, psychosocial and health outcomes, anthropometric measures, some clinical and metabolic indicators, and feasibility-related outcomes among people living with or beyond cancer.

In terms of the preferred nutrition-related outcome measures of those living with and beyond cancer, dietary intake and quality of life [31], in this review, dietary intake was assessed in 13 studies, with approximately 70% reporting positive changes following the intervention. Similarly, quality of life outcomes were evaluated in five studies, four of which reported significant improvements. These findings suggest that culinary nutrition interventions most consistently benefit outcomes that matter most to cancer survivors, indicating strong alignment with patient-identified needs and meaningful impact.

Overall, the reviewed evidence suggests that culinary nutrition interventions hold meaningful promise for supporting the health and wellbeing of people living with and beyond cancer [6,93,94,95]. Importantly, all studies were well-received, with high satisfaction, strong engagement, and few adverse effects, indicating that nutrition education programmes are both feasible and acceptable in survivorship care. The findings of this systematic review also reflect the broader needs of this population, emphasising the importance of supportive systems that facilitate sustainable dietary and lifestyle changes [9,10,13,14,96]. There is growing evidence that a healthy diet is linked to lower all-cause mortality in cancer survivors [6]; while many individuals understand healthy eating principles, making and maintaining meaningful changes, particularly after a cancer diagnosis, requires practical guidance, tailored support, and a structured environment to build skills. Culinary nutrition interventions address these needs by providing reliable, hands-on education that empowers individuals to apply recommendations in their daily lives [22,23]. This is especially relevant given that 88% of cancer survivors do not adhere to recommended healthy eating guidelines, highlighting a critical gap in post-treatment care [11]. Together, these findings highlight the need to integrate accessible, personalised, and supportive nutrition programmes into oncology care, recognising that sustained lifestyle change rarely happens in isolation—it requires coordinated support, community, and ongoing guidance.

Forty percent of the included studies incorporated elements of community-based participatory research (CBPR) [37,40,42,43,48,50,52,53]. Although it was not possible to quantify the level or impact of public and patient involvement (PPI) due to heterogeneity in both intervention design and participatory approaches, the inclusion of CBPR remains an important strength. People living with and beyond cancer have consistently highlighted the need for nutrition information and programmes to be tailored to their specific needs and lived experiences [9,14,15,17]. CBPR places community stakeholders at the centre of the research process and integrates experiential knowledge into the co-creation of health interventions alongside researchers and health professionals [97,98]. This collaborative approach enables the design of health promotion and prevention interventions that are responsive to the population’s context, preferences, and priorities. Importantly, CBPR facilitates the identification of both community-wide and subgroup-specific needs and supports a deeper understanding of how social, economic, and environmental determinants shape health behaviours and outcomes [97,98]. As such, CBPR-informed approaches may enhance the relevance, acceptability, and sustainability of culinary nutrition interventions in cancer care.

### 4.1. Quality of the Evidence

A key strength of the studies included in this review is that 88% of them drew on evidence-based nutrition recommendations and guidelines [35,36,37,38,39,40,41,42,43,44,46,47,49,50,51,52,53], and an equal proportion incorporated established behaviour-change theoretical frameworks to inform intervention design and content [35,36,37,38,40,41,42,43,44,46,47,48,50,51,52,53]. On the other hand, across the included studies, the overall risk of bias was substantial. Among the RCTs, most were rated as having some concerns [35,36,39,41,42,43], and three were rated as high risk [37,38,40]. Common issues included deviations from intended interventions, missing outcome data, and concerns regarding outcome measurement and selective reporting. The non-randomised studies demonstrated greater methodological limitations, with the majority, except for two studies [48,52], classified as having a critical risk of bias, primarily due to inadequate control of confounding [44,45,46,47,49,50,51,53].

### 4.2. Strengths and Limitations

One strength of the present review is that it is the first to comprehensively examine nutrition education interventions that include a culinary component for people living with and beyond cancer. While previous systematic reviews have evaluated the impact of nutrition education programmes [7,8,99,100,101,102], this is the first to focus specifically on culinary nutrition education interventions. This distinction is important, as cooking skills and practical culinary knowledge can enhance confidence, autonomy, and the capacity to adopt and sustain healthy eating behaviours [25]. This systematic review has several limitations that should be considered when interpreting the findings. Substantial heterogeneity across studies, including differences in sample size, intervention length, content and delivery format, and the range of outcomes assessed, limited the ability to compare results directly and quantify intervention effects or pool results in a meta-analysis. As described previously, the overall risk of bias was high, particularly among non-randomised studies, where inadequate control for confounding was common. Fifty percent of the selected studies targeted people with breast cancer [35,36,37,38,39,41,42,43,50], while 39% included participants with any cancer type [40,44,45,47,49,51,52]. Although this reflects the prevalence of breast cancer within the population [2], it nonetheless limits the generalisability of the findings to individuals with other cancer diagnoses. On the other hand, 15 studies (83%) were conducted in the post-treatment phase [35,36,38,40,41,42,43,44,45,46,47,48,49,50,51,52], which strengthens the relevance of the findings for individuals living beyond active cancer treatment.

### 4.3. Implications for Research and Practice

Although nutrition recommendations exist for people living with and beyond cancer, adapted and more practical strategies are needed to embed nutrition within oncology care and to support individuals in sustaining meaningful lifestyle changes over time [6]. This is particularly relevant given that the reviewed interventions were generally well tolerated and well accepted, with minimal reported adverse effects. At the same time, the limitations identified in this review emphasise the need for further high-quality research to better understand the role and effectiveness of these programmes, whether delivered alone or in combination with components such as physical activity or mental health support. Future research should extend beyond breast cancer to include a wider range of cancer types and more diverse populations. Greater inclusion of male participants, younger populations, and underrepresented groups is needed to improve the equity, relevance, and applicability of findings. A critical gap identified in this review is the lack of sustainability and long-term follow-up data. Future studies should evaluate the durability of dietary and health outcomes over time and assess whether culinary nutrition interventions support sustained long-term behaviour change.

## 5. Conclusions

This review summarises the effects of nutrition education programmes incorporating a culinary component in people living with and beyond cancer. The findings indicate that culinary nutrition interventions, both standalone and those combined with physical activity or mental health components, are generally beneficial, demonstrating improvements across most outcome areas, especially outcomes preferred by the cancer community—dietary intake and quality of life [31]. While the review cannot determine the precise magnitude of these effects, our findings summarise the ongoing research in this field and help understand their potential, direction, and scope, highlighting important areas for further investigation. The emerging evidence aligns with current oncology nutrition guidelines and highlights the potential of culinary nutrition programmes as supportive, behaviour-focused strategies that can complement clinical care and improve survivorship outcomes.

## Figures and Tables

**Table 1:** Summary characteristics of each study.

First Author (Year) Programme Place	Participants, Cancer Type, Timepoint, Sample, Gender, Mean Age	Study Design	Nutrition Content	Delivery	Design: (a) Resources; (b) Theoretical Framework; (c) Co-design	Outcome Measures
Randomised Controlled Trials (RCT)						
Greenlee et al. 2015 [43] “¡Cocinar Para Su Salud!” USA	Hispanic adult women Breast cancer Posttreatment Int n = 34 Control n = 36 100% female 56 ± 9.5 yo	RCT Nutrition single component Quantitative	Nutrition education roundtables, cooking lessons and shopping trips: healthy eating, food groups and nutrients, cooking skills, healthy and budget friendly shopping, meal planning and preparation, portion size, meal sharing, food taste, food labels Culturally tailored	Group In person 9 sessions 12 weeks 24 h total Monthly calls	(a) AICR and ACS Guidelines [54,55]. (b) Contento’s Model [56], Social Cognitive Theory [57], The Transtheoretical Model of Health Behaviour Change [58] (c) Community Interviews and Focus Groups.	Primary—Dietary Intake Secondary—Anthropometric Timepoints M3 and M6
Greenlee et al. 2016 [42] “¡Cocinar Para Su Salud!” USA	Hispanic adult women Breast cancer Posttreatment Int n = 34 Control n = 36 100% female 56 ± 9.5 yo	RCT Nutrition single component Quantitative	Nutrition education roundtables, cooking lessons and shopping trips: healthy eating, food groups and nutrients, cooking skills, healthy and budget friendly shopping, meal planning and preparation, portion size, meal sharing, food taste, food labels. Culturally tailored	Group In person 9 sessions 12 weeks 24 h total Monthly calls	(a) AICR and ACS Guidelines [54,55]. (b) Contento’s Model [56], Social Cognitive Theory [57,59], The Transtheoretical Model of Health Behaviour Change [58]. (c) Community Interviews and Focus groups.	Primary—Dietary Intake. Secondary—Anthropometric, Clinical and Metabolic Timepoint M12
Greenlee et al. 2024 [41,60] “Cook and Move for Your Life” USA	Adult women Early stage breast cancer Posttreatment Int n = 38 Control n = 36 100% female 58 ± 10 yo	RCT Multicomponent Nutrition and Physical Activity Quantitative	Nutrition and physical activity education, hands-on skill building culinary cooking lessons: healthy eating, food groups and nutrients, cooking skills, meal planning and preparation, portion size, meal sharing, food labels, eating out.	Group Online Text messages Newsletters 12 sessions 1.5 h/session 6 months 18 h total	(a) AICR and ACS Guidelines [54,55]. (b) Social Cognitive Theory [61,62], Self-Determination Theory [63] (c) None Reported.	Primary—Feasibility. Secondary—Effectiveness (Dietary Intake, Anthropometric, And Psychosocial) Timepoint M6
Miller et al. 2020 [40] “Coping with Cancer in the Kitchen” USA	Adults Any cancer types Posttreatment Int = 27 Control = 27 92% female 61 ± 10.5	RCT Nutrition single component Quantitative	Nutrition education, group learning and support, cooking demonstrations, eating together, goal setting: plant-based healthy eating, food groups and nutrients, cooking skills, meal planning and preparation, portion size, meal sharing, eating together, food taste.	Group In-person 8 sessions 1.5 h/session 9 weeks 12 h total	(a) AICR Guidelines [55]. (b) Social Cognitive Theory [64], The Transtheoretical Model of Health Behaviour Change [58,65], Other behaviour change techniques [66,67,68], Smart Goal Setting. (c) Co-design With Stakeholders.	Primary—Nutrition Knowledge. Secondary—Dietary Intake, Psychosocial Outcomes. Timepoints WK9 and WK15
Morato-Martínez et al. 2021 [39] Spain	Adult women Breast cancer During treatment Int = 32 Control = 33 100% female 50 ± 9.43 yo	RCT Multicomponent Nutrition and Physical Activity Quantitative	Nutrition and physical activity education, individualised diets: obesity-related complications, healthy eating, food groups and nutrients, cooking skills, meal planning and preparation, portion size, shopping, food labels, eating out, food myths, anxiety management.	Group In-person 5 sessions 1 h/session 6 months 5 h in total 4 PA sessions per week	(a) Spanish Society of Community Nutrition Guidelines [69], WHO Physical Activity Guidelines [70]. (b) None Reported. (c) None Reported.	Primary—Anthropometric Secondary—Physical Activity, Dietary intake, Clinical and Metabolic Timepoints WK24 and WK48
Parekh et al. 2018 [38] “The HEAL-BCa Study” USA	Adult women Breast cancer Posttreatment Int = 31 Control = 28 100% female 58 ± 10.3 yo	RCT Multicomponent Nutrition and Physical Activity Mixed methods	Nutrition education, interactive physical activity education sessions, cooking demonstrations, hands-on culinary cooking: cancer and diet, healthy eating, food groups and nutrients, cooking skills, meal planning and preparation, portion size, food shopping, food labels, pathophysiology of breast cancer and modifiable risk factors.	Group In-person 6 sessions 2 h/session 3 months 12 h total	(a) AICR and ACS Guidelines [54,55]. (b) Social Cognitive Theory [71]. (c) None Reported.	Dietary Intake, Anthropometric, Feasibility Qualitative Timepoint M3
Sheean et al. 2021 [37] “Every day counts” USA	Adult women Metastatic breast cancer During treatment Int = 17 Control = 18 100% female 55 ± 12.3 yo	RCT Multicomponent Nutrition and Physical Activity Quantitative	Nutrition education written materials, hands-on culinary cooking, supervised physical activity: healthy eating, food groups and nutrients, plant-based cooking skills, meal planning and preparation, portion size, barriers, behaviour change strategies. Tailored physical activity guidance.	Group In-person and telephone 12 weeks, 12 weekly phone calls and text messages 4 PA sessions 3 hands-on culinary cooking sessions	(a) ACS Guidelines [54]. (b) Social Cognitive Theory. (c) Community Advisory Board.	Dietary Intake, Anthropometric, Psychosocial, Symptom Management, Clinical and Metabolic. Qualitative Timepoints WK12
Sheppard et al. 2016 [36] “The Stepping STONE study” USA	African American adult women Breast cancer Posttreatment Int = 15 Control = 16 100% female 55 ± 9.8	RCT Multicomponent Nutrition, Physical Activity and Behaviour Change Mixed methods	Nutrition and PA education, cooking demonstrations, supervised PA, interviews: healthy eating, food groups and nutrients, cooking skills, meal planning and preparation, portion size, barriers, behaviour change strategies, survivorship concerns. Culturally tailored	Group In-person and telephone 12 weeks 6 sessions 1.5 h/session 6 phone motivation interviews	(a) ACS Guidelines [54]. (b) Theory of Planned Behaviour [72] and Social Cognitive Theory [73]. (c) None Reported.	Dietary Intake, Physical activity, Anthropometric, Psychosocial, Clinical and Metabolic, Intervention Satisfaction. Qualitative Timepoint WK12
Zuniga et al. 2019 [35] USA	Adult women Breast cancer Posttreatment Int = 60 Control = 65 100% female 57 ± 9.3 yo	RCT Nutrition single component Quantitative	Nutrition education, cooking demonstrations, newsletters, motivation interviews: diet-cancer relationship, anti-inflammatory Mediterranean healthy eating, food groups and nutrients, cooking skills, meal planning and preparation, recipes, portion size, food taste, food shopping, barriers, behaviour change strategies, goal setting. Tailored anti-inflammatory dietary prescription.	Group In-person and telephone 6 sessions Session duration not specified 6 months 6 phone motivation interviews 6 Newsletters	(a) Mediterranean Diet Guidelines [74,75,76]. (b) Stages of Change [73]. (c) None Reported.	Dietary Intake Timepoint WK12
Non-randomised Controlled Trials (Non-RCT)						
Allen-Winters et al. 2020 [53] “Eat to live” USA	Caucasian adults Head and Neck cancer patients and caregivers 3 patients-1 care giver 33% female 67 yo	Non-RCT Nutrition single component Quantitative	Nutrition education, cooking sessions adapted for H&N cancer patients: healthy eating, food groups and nutrients, cooking skills, meal planning and preparation, recipes, portion size, food taste, food shopping, barriers, eating together, behaviour change	Group In-person 3 sessions 2 h/session 3 months	(a) WHO’s Nutrient Intake Goal [77]. (b) The Transtheoretical Model of Health Behaviour Change. (c) Pre-Intervention Surveys Informed Recipe Design.	Dietary Intake, Behaviours and Preferences, Sensory Timepoint M6
Barak-Nahum et al. 2016 [52] Israel	Adults Any cancer types During and posttreatment Int = 96 Control = 88 93% female 58 ± 9.7 yo	Non RCT Multicomponent Nutrition and Psychosocial education Mixed methods	Core element was hands-on cooking, nutrition education, psychosocial education, eating and chatting together: Mediterranean healthy eating, food groups and nutrients, cooking skills, meal planning and preparation, recipes, portion size, food taste, barriers, eating together, psychosocial aspect of eating, behaviour change, mindful eating, improvisation, creativity. Culturally tailored	Group In-person 10 sessions 2 h/session 10 weeks	(a) AICR Guidelines [55]. (b) Other Behaviour Change Techniques [78]. (c) Participants Brainstormed to Define Themes.	Psychosocial Timepoint WK10
Golubić et al. 2018 [51] Lifestyle180^®^ USA	Adults Any cancer types Posttreatment n = 58 No control 76% female 64 ± 8.7 yo	Non RCT Multicomponent Nutrition, Physical Activity and Mental Health Quantitative	Lifestyle intervention added to treatment plans, education on nutrition, physical activity, stress management, cooking skills: Mediterranean healthy eating, food groups and nutrients, cooking skills, meal planning and preparation, recipes, portion size, food taste, food shopping, food labels, lifestyle behaviour change. As well as physical activity and stress management skills	Group In-person 12 sessions 4 h/session 6 weeks Follow-up sessions (week 10, 18 and 30) 30 newsletters Buddy system	(a) Mediterranean Diet Guidelines [79]. (b) Lifestyle Behaviour Change Framework [80]. (c) None Reported.	Dietary Intake, Anthropometric, Clinical and Metabolic, Psychosocial. Timepoints M12
Huang et al. 2023 [50] “SOAR” USA	Adult women Breast cancer Posttreatment Int = 102 No control 98% female 68% over 55 yo	Non RCT Multicomponent Nutrition, Physical Activity, and Mental Health Quantitative	Virtual teaching kitchen with culinary-cooking demonstrations delivered by a culinary expert, followed by dietitian-led discussion at each session, nutrition education, mental health, exercise and behaviour change: Mediterranean healthy eating, food groups and nutrients, cooking skills, recipes, food shopping, mindful eating, emotional health, yoga and meditation, art, life after cancer treatment.	Group Virtual 9 sessions Duration of sessions not specified 9 weeks	(a) AICR Guidelines [55], Mediterranean Diet Guidelines [81] (b) Other Behaviour Change Techniques [82]. (c) Participants Identified Learning Objectives.	Psychosocial Timepoint WK9
Jackson et al. 2024 [49] USA	Adult Any cancer types Posttreatment n = 24 No control 100% female 61.5 yo median (19.5 IQR)	Non RCT Nutrition single component Quantitative	Virtual cooking class, nutrition education with extra education materials provided: healthy eating, focus on anti-inflammatory diet, food groups and nutrients, recipes, cooking skills.	Group Virtual 1 session	(a) AICR Guidelines [55], Mediterranean Diet [83,84]. (b) None Reported. (c) None Reported.	Dietary Intake, Psychosocial. Timepoint M1
Kondo et al. 2023 Japan	Adults Digestive cancer During—Post surgery Int = 13 Control = 11 83% female 68 yo median	Non RCT Nutrition single component Retrospective Quantitative	Hands-on cooking, disease management, mental health: cooking skills, post-surgery management, emotional wellbeing.	Individual In-person One session 1.5 h	(a) None Reported. (b) Experiential Learning Theory [85]. (c) Participants Decided on Recipes.	Psychosocial. Timepoint Before Hospital Discharge.
Pritlove et al. 2020 [47] “Cooking for vitality” Canada	Adults experiencing cancer-related fatigue Patients and caregivers Any cancer types Posttreatment n = 58 No control 88% female 58 ± 12.3 yo	Non RCT Nutrition single component Mixed methods	Hands-on cooking, education on nutrition and cancer-related fatigue (CRF): healthy eating for CRF, food groups and nutrients, meal planning and preparation, recipes, cooking skills, barriers, goal setting.	Group In-person 2 sessions 1.5 h/session 6 support emails 6 weeks	(a) Fatigue Reduction Diet In Breast Cancer Survivors [86]. (b) Social Cognitive Theory, Social Learning Theory [64], Experiential Learning Theory [85], Other Behaviour Change Techniques [87,88]. (c) None Reported.	Feasibility, Psychosocial Timepoint WK6 and WK18
Pritlove et al. 2024 [46] “EDIBLE” Canada	Adult women Non-metastatic Gynaecological cancer and caregivers Post-radiation treatment Int = 53 patients No control 100% female 63.1 ± 10.5 yo	Non RCT Nutrition single component Mixed methods	Nutrition education, hands-on cooking: healthy eating, food groups and nutrients, cancer side effects, self-management, nutrition counselling, cooking skills, recipes, shopping lists.	Group In-person 2 sessions 1.5 h/session 7 weeks	(a) Nutrition, Cancer, and Gastrointestinal Complications Guidelines. (b) Social Cognitive Theory and Social Learning Theory [64], Experiential Learning Theory [85], Other Behaviour Change Techniques [87,88]. (c) None reported.	Feasibility and Psychosocial Qualitative. Timepoint WK9 and WK12
Raber et al. 2022 [45] “Cooking after cancer” USA	Adults Any cancer types Posttreatment n = 20 No control Age and gender not reported	Non RCT Nutrition single component Quantitative	Food prep demonstration and hands-on cooking, nutrition education: plant-based healthy eating, food groups and nutrients, food safety, food and meal prep, recipes, food labels, cooking skills, specific needs for cancer survivors.	Group In-person 6 sessions 1.5 h/session 6 weeks	(a) The Happy Kitchen’s Standard Community Cooking Class Curriculum, AICR Guidelines [55]. (b) None Reported. (c) None Reported.	Psychosocial Timepoint WK6
Spees et al. 2019 [44] USA	Adults Any cancer types Posttreatment n = 35 83% female 58 yo	Non RCT Multicomponent Nutrition and Physical Activity Mixed methods	Hands-on vegetable gardening education, nutrition and lifestyle education, cooking demonstrations: plant-based healthy eating, food groups and nutrients, health lifestyle, cooking skills, using garden veg, recipes, food and meal prep, physical activity, coaching, targeted information for cancer side effects, supportive technologies (pedometer, resources website)	Group In person Virtual coaching 24 garden sessions 24 education sessions 6 months	(a) AICR Guidelines [55], AHA/ACC Guidelines [89], Garden-Based Lifestyle Interventions [90]. (b) Self-Determination Theory [91]. (c) None Reported.	Feasibility, Dietary Intake, Physical Activity, Anthropometric, Clinical and Metabolic, Psychosocial. Qualitative Timepoint M6

AICR: American Institute of Cancer Research; ACS: American Cancer Society; WHO: World Health Organisation; AHA/ACC: American Heart Association/American College of Cardiology; and yo: years old.

**Table 2:** Outcomes measured, summary of results and risk of bias of each study.

Study Details	Outcomes Measures	Outcome Measures—Results	Risk of Bias *
Randomised Controlled Trials			
Greenlee et al. 2015 [43] Nutrition single component RCT Quantitative USA	Primary—Dietary intake (calorie, fruit and veg, fat). Secondary—Anthropometric (weight, BMI, waist/hip ratio). Timepoints M3 and M6	Dietary Intake 24 h dietary recall and questionnaires. Significant increase in daily F/V servings (+2, *p* = 0.005), with changes maintained at M6. Significantly lower percent calories from total fat (−7.1%, *p* = 0.01) and saturated fat (−3.8%, *p* < 0.001) at 3 months. Anthropometrics No significant change in weight (−2.5%, *p* = 0.22). Marginally significant waist circumference reduction at M6 (−1.6 cm, *p* = 0.05).	Some concerns (D2.1, D2.2, D4, D5)
Greenlee et al. 2016 [42] Nutrition single component RCT Quantitative USA	Primary—Dietary intake (calorie, fruit and veg, fat). Secondary—Anthropometric (weight, BMI, waist/hip ratio). C&M (carotenoids, glycaemic markers, inflammation, DNA methylation). Timepoint M6 and M12 follow up.	Dietary Intake Maintained significant increases in mean daily F/V servings (+2.3, *p* < 0.01), maintained significantly higher intake of citrus fruit, dark-green vegetables, and deep-yellow vegetables. Non-significant change in the % of calories from total fat (−3.1%, *p* = 0.29) and saturated fat (−1.6%, *p* = 0.1) at M12. Anthropometric Non-significant changes in weight (−3.1%, *p* = 0.5), BMI (−2.8%, *p* = 0.59), and maintained waist circumference. Clinical and Metabolic Measures Significant increase in plasma lutein at M6 (+13.8% *p* < 0.01) and M12 (+20.4%, *p* < 0.01). Glycaemic markers—At M6, non-significant changes in fasting glucose, improved insulin, and insulin sensitivity (HOMA-IR). Values not reported. No change at M12. Inflammation markers—non-significant change in IL1a, IL6, IL10, TNFa, and CRP-hs at M6 (values not reported). At M12, the level of inflammatory markers increased in both groups, but the intervention group showed non-significant changes in GM-CSF, IL-6, IL-8, and TNF-a. DNA methylation—At M6, non-significant change in global DNA methylation (+0.9% vs. *p* = 0.56); and maintained a borderline significant change at 12 months (+0.8%, *p* = 0.06).	Some concerns (D2.1, D2.2, D5)
Greenlee et al. 2024 [41] Multicomponent RCT Quantitative USA	Primary— Feasibility (accrual rate, adherence, retention, and acceptability). Secondary— Effectiveness: Dietary intake, Anthropometric (BMI), Psychosocial (Anxiety scores). Timepoint M6	Dietary Intake 24 h dietary recall: Significant increase in F/V serving per day (+1.5, *p* = 0.007), more servings of veg/day (+1.1, *p* = 0.006), reduced daily calorie intake (−170 kCal, *p* = 0.026), reduced saturated fat intake (−4.6 g, *p* = 0.006) and total fat (−10 g, *p* = 0.048). Anthropometric No significant changes in BMI (−0.24, *p* = 0.66) or weight (−0.51, *p* = 0.63). Psychosocial No significant changes in anxiety scores (+2.53, *p* = 0.1) or physical functioning scores (−0.56, *p* = 0.12) (PROMIS). Feasibility Attendance was high (84% high-dose; 94% low-dose). Low-dose participants responded to more texts (*p* = 0.03), while high-dose participants accessed the website more often (84% vs. 67% *p* = 0.08). Retention was excellent (92–97%). 90% of high-dose participants found sessions more helpful (*p* = 0.001).	Some concerns (D1, D2.1, D2.2, D4)
Miller et al. 2020 [40] Nutrition single component RCT Quantitative USA	Primary— Nutrition knowledge, confidence and skills regarding plant-based diets. Secondary— Dietary (dietary intake, perceived barriers to eating more fruit and veg). Psychosocial (QoL, psychological distress, fatigue, emotional support, perceived control over cancer) Timepoints WK9 and WK15	Dietary Intake Significantly improved plant-based nutrition knowledge (WK9&15), confidence (WK9&15) and skills (WK9&15). Scale points increase of +0.8/+06, +0.6/+0.7 and +0.6/+04 respectively (*p* < 0.05) (NCIDSQ). No-significant change in F/V intake and whole grains. No significant differences in perceived barriers to eating more F/V. Psychosocial Quality-of-life outcomes (FACT-G7), psychological distress (PHQ4), and fatigue (FSI) were not significantly different. Perceived control was stable across groups (SOC8).	High (all domains)
Morato-Martínez et al. 2021 [39] Multi-component RCT Quantitative Spain	Primary—Anthropometric (BMI, waist circumference, body weight, skinfold thickness measurements) Secondary—Physical Activity, Dietary intake (food frequency), C&M (blood pressure, HR, lipid profile) Timepoints WK24 and WK48	Dietary Intake Non-significant differences, both groups increased daily intake of grains, fruits, oily fish, dairy products, and oils, and reduced consumption of red meat and sweets via 72 h dietary recall and food intake frequency questionnaire. Anthropometric Significant changes in body weight (−1.87 kg, *p* = 0.005) and BMI (−0.61 kg/m^2^, *p* = 0.011). Non-significant differences between groups in waist circumference or in skinfold thickness. Clinical and Metabolic No significant changes were observed in blood pressure or heart rate. Significant reduction in LDL cholesterol in the intervention group at WK24 and WK48 (−35.29 mg/dL and −12.5 mg/dL, *p* = 0.003), and total cholesterol at WK24 and WK48 (−29.29 mg/dL and −32.92 mg/dL, *p* = 0.005). No changes were reported in triglycerides, glucose, HDL cholesterol, or protein. Physical activity Intervention group was significantly less sedentary at WK48 (−2.7 h, *p* = 0.04) Both groups showed increased physical activity at 1 year, with no group differences.	Some concerns (all domains)
Parekh et al. 2018 [38] Multicomponent RCT Mixed methods USA	Dietary Intake (nutrition knowledge, F/V, health literacy), Anthropometric (BMI). Feasibility (recruitment, acceptability) Qualitative. Timepoint M3	Dietary Intake High baseline nutrition/health literacy except for food portions (NLit-BCa/NVS). Similar baseline dietary patterns across groups (Block screener). Alcohol intake declined in both groups; no significant intervention effect. Anthropometric BMI decreased slightly in controls and more in the intervention group (−0.69 kg/m^2^, *p* = 0.044); significant only in age-adjusted model. Intervention associated with lower risk of higher BMI (RR = 0.52; 95% CI 0.28–0.99). Feasibility Recruitment goals were met within 6 months. Among intervention participants with complete data, 52% attended all sessions, 32% attended five sessions, and 16% attended four. Qualitative findings indicated high acceptability, helpful hands-on cooking sessions, strong group support, suggestions for more tailored resources and continued free access, and interest in booster sessions.	High (all domains)
Sheean et al. 2021 [37] Multicomponent RCT-Mixed USA	Dietary Intake (energy, intake of wholegrains, fruit and veg, processed and red meat, alcohol), Anthropometric (BMI, body composition, hand-grip), Psychosocial (QoL, depression, anxiety, perceived stress), Symptoms management (breast cancer, endocrine and fatigue symptoms), C&M (respiratory capacity). Qualitative Timepoints WK12	Dietary Intake. Dietary changes were not evident across groups due to high baseline adherence. Anthropometric No changes in BMI or body composition. Visceral fat decreased in all groups (−89 g, *p* < 0.05). Non-significant changes for sarcopenia (20% vs. 14%; *p* = 0.938). Handgrip strength improved in intervention (+4.9 kg, *p* = 0.002). Psychosocial Significant increases in QOL scores (FACT General) between groups (+4.9, *p* = 0.003) and for women in the intervention group (*p* = 0.001), driven by improvements in physical (+ 1.6, *p* = 0.003), emotional (+1.4, *p* = 0.01) and functional wellbeing (+1.2, *p* = 0.06). Depression decreased more in the intervention vs. control (−0.9, *p* = 0.03) (HADS). Anxiety decreased in both groups, only significant in control group (between groups: −1.2, *p* = 0.021; intervention and control: −1.1, *p* = 0.085 vs. −1.3, *p* = 0.024, respectively). Perceived stress significantly declined in both groups (intervention and control: −3.1, *p* = 0.003 vs. −2.5, *p* = 0.001 respectively) but larger change in intervention group (−2.8, *p* = 0.001). Symptoms Management Intervention group improved breast cancer (+6.8, *p* = 0.001) and endocrine (+ 8.7, *p* = 0.002) symptoms. Fatigue also improved in the intervention group (+1.6, *p*= 0.037). Clinical and Metabolic Spare respiratory capacity increased (+69.7%, *p* = 0.24), indicating potential cellular changes in muscle function. Qualitative Recommendations included the desire for a longer intervention with additional support.	High (D2.1, D2.2, D4, D5)
Sheppard et al. 2016 [36] Multicomponent RCT-Mixed USA	Primary—Dietary Intake (daily energy intake, % energy from fat, and grams of fat and fibre), Physical activity, anthropometric weight, BMI, waist and hip circumference), Psychosocial (self-efficacy, satisfaction), C&M (VO_2_ max), Intervention satisfaction. Qualitative Timepoint WK12	Dietary Intake Non-significant changes in total energy intake, total fat, % energy from fat, and fibre intake in intervention group. Physical Activity Total physical activity levels increased in the intervention group 3.6-fold. Anthropometric Non-significant changes in weight, BMI, and lower waist/hip ratio in the intervention group. Psychosocial. Self-efficacy, participants had high perceived control in achieving and maintaining their dietary and physical activity goals. Overall satisfaction was high (86%). Clinical and Metabolic VO_2_ max changes were not statistically significant. Qualitative Participants suggested including more opportunities to enhance interaction with other group members and the study staff and tailoring the programme to a greater extent to their condition.	Some concerns (All domains)
Zuniga et al. 2019 [35] Nutrition single component RCT Quantitative USA	Dietary intake (Mediterranean diet intake, spices and herbs intake, calorie intake). Timepoint M6	Dietary Intake Mediterranean diet adherence increased in the intervention vs. control group (+22.5%, *p* < 0.001), with greater adherence to fish/shellfish, reduced red meat, and limited sweets. Use of spices/herbs also increased more in the intervention group (+146.2%, *p* < 0.001), including higher intake of cinnamon, turmeric, garlic, ginger, black pepper, and rosemary. Calorie intake decreased in intervention vs. control (−195.5 kcal, *p* = 0.045); no differences for macronutrients, sodium, fibre, or fruit/vegetable intake.	Some concerns (All domains)
Non-randomised controlled trials			
Allen-Winters et al. 2020 [53] Nutrition single component Non-RCT Quantitative USA	Dietary intake, behaviours and preferences (eating habits, dietary preferences, cooking habits, meal planning habits, cooking perception), Sensory (taste function) Timepoint M6	Dietary Intake, behaviours and preferences Improvements in dietary choices, (limited small sample size) despite disruptions of taste sensations. Healthful eating scores increased modestly from start to finish of the class (1.5 to 1.7 on a 3-point scale).	Critical (not eligible for RoB assessment)
Barak-Nahum et al. 2016 [52] Multicomponent Non-RCT Mixed Israel	Psychosocial (QoL, subjective wellbeing, Intuitive eating). Timepoint WK10	Psychosocial Overtime, intervention group showed higher health-related quality of life (SF12) (+13.23, *p* = 0.005) and lower subjective wellbeing’s negative affect (+28, *p* < 0.001), and positive affect (40.57, *p* < 0.001). Intuitive eating increased (IES) over time (*p* < 0.001): unconditional permission to eat (+25.1), eating for physical rather than emotional rea-sons (+22.9), and reliance on hunger and satiety cues (+37.3). In the wait-list group, no changes occurred in unconditional permission to eat or eating for physical reasons, and reliance on hunger cues decreased (*p* = 0.012).	Moderate (D1, D6)
Golubić et al. 2018 [51] Multicomponent Non-RCT Quantitative USA	Dietary intake (food diary), Anthropometric (BMI, waist circumference), C&M (cardiometabolic—resting heart rate, blood pressure; glycaemic and lipid profiles; inflammation US-CRP), Psychosocial (QoL, perceived stress, depression). Timepoints M12	Anthropometrics BMI decreased significantly by 2.4 kg/m^2^ (−7%, *p* < 0.001), lost 7% body weight (*p* < 0.001) and 6.6 cm waist reduction (*p* < 0.001). Clinical and Metabolic Blood pressure changes were not significant, though 30% participants reduced BP medications while 15% increased their medication. Increased HDL (+3.3 mg/dL, *p* < 0.05), decreased triglycerides (−23.0 mg/dL, *p* < 0.01), ultrasensitive CRP (−1.3 mg/L, *p* < 0.01), fasting insulin (−4.2 mU/mL, *p* < 0.05), and HOMA-IR (−1.5, *p* < 0.01). Lipid-lowering medication use decreased (12% reduced vs. 3% increased). Non-significant changes in LDL (−6.2 mg/dL *p* = 0.06) and fasting glucose (−8.8 mg/dL, *p* = 0.17). Psychosocial Significant decrease in perceived stress (PSS-4) (−20%, *p* < 0.05). Physical (+62%, *p* < 0.01), mental (+51%, *p* < 0.05) and overall health quality of life (+54%, *p* < 0.001) scores significantly increased (VR-12). Depression unchanged (CES-D 10). Dietary Intake Despite limited complete data (for dietary intake only, ~10%), weekly dietary fat intake decreased significantly (n = 6, *p* = 0.007), and fruit/vegetable/fibre servings showed non-significant changes (*p* = 0.13).	Critical (not eligible for RoB assessment)
Huang et al. 2023 [50] Multicomponent Non-RCT Quantitative USA	Psychosocial (mindful eating) Timepoint WK9	Psychosocial Significant increase in Mindful Eating Questionnaire summary scores (scale mean change 0.12, *p* < 0.001); all subscales improved except for the distraction subscale.	Critical (not eligible for RoB assessment)
Jackson et al. 2024 [49] Nutrition single component Non-RCT Quantitative USA	Dietary intake (dietary inflammatory index) Psychosocial (cognitive function) Timepoint M1	Dietary Intake Diet History Questionnaire (DHQIII) and Dietary Inflammatory Index (DII): The Energy-Adjusted Dietary Inflammatory Index (E-DII) decreased, indicating a more anti-inflammatory diet (−0.4, *p* = 0.005). Psychosocial Cognitive outcomes improved significantly, including perceived cognitive impairment, comments from others about cognition, and cognition-related quality of life (all *p* < 0.001). There were significant increases in cognition (FACT-Cog), including perceived cognitive impairment (COG-PCI, Δmedian −38 *p* < 0.001), comments from others (COG-OTH, Δmedian −16, *p* < 0.001), and quality of life (COG-QOL, Δmedian −10, *p* < 0.001). A change in calories was a significant predictor of a change in perceived cognitive ability (COG-PCA) after. No significant correlations between E-DII and cognitive variables at follow-up.	Critical (not eligible for RoB assessment)
Kondo et al. 2023 [48] Nutrition single component Non- RCT Quantitative Japan	Psychosocial (anxiety and depression, daily living activity) Timepoint before hospital discharge	Psychosocial Improvements on the Hospital Anxiety and Depression Scale (HADS) were observed in the cooking group for both anxiety (−4 scale points, *p* = 0.035) and depression (−6 scale points, *p* = 0.045). The Functional Independence Measure improved in both groups (*p* = 0.008).	Moderate (D1, D3, D6, D7)
Pritlove et al. 2020 [47] Nutrition single component Non-RCT Mixed Canada	Feasibility (recruitment, retention, adherence, qualitative feedback). Psychosocial (symptoms management, fatigue, energy levels, disability) Timepoint WK6 and WK18	Feasibility Recruitment (70%) and retention (72%) rates, together with the qualitative findings, support the feasibility of the intervention. Acceptability and satisfaction were also high. Symptoms management Fatigue scores (FACT-F) improved significantly from baseline to T1 and T2 (+5 and +7.75, *p* < 0.001). Disability (WHO-DAS 2.0) decreased from baseline to T2 (−2.43, *p* = 0.006). Energy levels (Profile of Mood States) increased from baseline to T1 (+1.46, *p* = 0.018) and T2 (+1.63, *p* = 0.013). Cooking confidence in managing fatigue improved from baseline to T1 and T2 (+7.74, +11.07, *p* < 0.001).	Critical (not eligible for RoB assessment)
Pritlove et al. 2024 [46] Nutrition single component Non-RCT Mixed Canada	Feasibility (recruitment, retention, adherence, qualitative feedback) Psychosocial (symptom management, nutrition knowledge, and self-efficacy) Qualitative. Timepoint WK9 and M3	Psychosocial Symptoms management assessment via the IBDQ disease-specific health-related QoL questionnaire reported significant improvements (*p* = 0.003) in all dimensions: bowel (*p* = 0.004), systemic (*p* = 0.01), social *p* = 0.03), and emotional (*p* = 0.04) at M3. Both nutrition knowledge and self-efficacy significantly improved across all areas at M3 (*p* < 0.001). Feasibility and qualitative Qualitative interviews supported strong perceptions of intervention feasibility; however, the recruitment (32%) and retention (72%) rates were modest, indicating that alternate formats for programme delivery may be needed. High ratings on measures of satisfaction and utility, with program recommendations being increased in-class sessions and program expansion.	Critical (not eligible for RoB assessment)
Raber et al. 2022 [45] Nutrition single component Non-RCT Quantitative USA	Psychosocial (nutrition knowledge and food behaviour) Timepoint WK6	Psychosocial Food knowledge and behaviours, assessed via Healthy Cooking Questionnaire (HCIQ2), no significant difference in baseline vs. post intervention Healthy Cooking Index (HCI) scores.	Critical (not eligible for RoB assessment)
Spees et al. 2019 [44] Multicomponent Non-RCT Mixed USA	Feasibility (recruitment, retention, adherence, qualitative feedback), Dietary Intake (food frequency), Physical Activity (steps), Anthropometric (weight, BMI, waist circumference) C&M (blood pressure, carotenoids, lipid profile, average glucose, inflammatory markers, hormone profile), Psychosocial (QoL, behaviour risk, self-efficacy). Qualitative Timepoint M6	Dietary Intake Significant changes in dietary intake, Healthy Eating Index total score increased by 5.2 points (*p* = 0.006). Daily energy intake decreased by 250 kcal (*p* = 0.012). Vegetable intake increased by 1.05 servings (*p* < 0.001); fruit intake increased by 0.41 servings (*p* = 0.022). Added sugar intake decreased by 2.37 tsp (*p* = 0.036). Physical Activity Physical activity increased, with mean daily steps rising by 18.9% (*p* = 0.033). Anthropometric Significant reductions (all *p* < 0.001) occurred in body weight (−3.9 kg), BMI (−1.5 kg/m^2^), and waist circumference (−5.5 cm). Clinical and metabolic Significant decrease in systolic blood pressure (−9.5 mmHg, *p* = 0.006). Total cholesterol and triglycerides significantly decreased (6% and 14%, *p* = 0.04 and *p* = 0.01, respectively). No significant changes for HDL and LDL cholesterol. Inflammatory markers declined, hs-CRP (−28%, *p* = 0.004) and IGFBP-3 (−5%, *p* = 0.005); No significant changes for average blood glucose (HbA1c) and insulin. Carotenoid status improved, with total dietary carotenoids increasing 66% (*p* < 0.001) and total plasma carotenoids increasing 35% (*p* < 0.001). Individual plasma carotenoids also increased (alpha-carotene (*p* < 0.001), beta-carotene (*p* < 0.001), lycopene (*p* = 0.017). Skin carotenoids increased (*p* = 0.015) and correlated strongly with plasma carotenoids. Psychosocial Overall quality of life improved significantly (+16.07 points, *p* = 0.004), with gains across physical, psychological, and spiritual wellbeing. Total self-efficacy showed no significant changes. Participants reported reduced use of medications/supplements and fewer barriers to healthy eating (e.g., cost, food preferences, preparation knowledge, access, and willpower). Feasibility and qualitative Education session attendance was high (90% overall; 84% mean session attendance). Participants attended 59% of harvest weeks; with 52% completing all weeks and 90% ≥ 80% of weeks. Most participants used the web portal (90%) and tele-motivational interviewing (59%). Email was the primary mode (71%), followed by phone (57%) and text (10%). High acceptability was reported, 93% rated the programme and harvesting as “excellent/very good.” Group education was viewed as most effective (55%), followed by harvesting (34%) and tele-motivational interviewing (18%). Most participants reported positive health impacts (97%) and a strong sense of community (93%), and 97% would recommend the program. Nearly all agreed it improved dietary patterns (97%) and physical activity (93%). All participants planned to use the information for future health decisions.	Critical (not eligible for RoB assessment)

* Risk of Bias Assessment Overall Score (affected domains—D1 to D7). See Supplementary Data for details, RoB Assessment.

## Data Availability

All data supporting the findings of this study are contained within the article and its Supplementary Materials.

## Supplementary Materials

The following supporting information can be downloaded at: https://www.mdpi.com/article/10.3390/curroncol33020076/s1, Figure S1: Search criteria. Supplementary Data: RoB Assessment.
